# CDE-1 suppresses the production of risiRNA by coupling polyuridylation and degradation of rRNA

**DOI:** 10.1186/s12915-020-00850-z

**Published:** 2020-09-04

**Authors:** Yun Wang, Chenchun Weng, Xiangyang Chen, Xufei Zhou, Xinya Huang, Yonghong Yan, Chengming Zhu

**Affiliations:** 1 National Science Center for Physical Sciences at Microscale Division of Molecular & Cell Biophysics, School of Life Sciences, University of Science and Technology of China, Hefei, 230027 Anhui People’s Republic of China; 2 School of Bioengineering, Huainan Normal University, Huainan, 232038 Anhui People’s Republic of China; 3 National Institute of Biological Sciences, Beijing, 102206 People’s Republic of China

**Keywords:** rRNA, siRNA, Argonaute, risiRNA, CDE-1, SUSI-1, Uridylation, Inheritance

## Abstract

**Background:**

Modification of RNAs, particularly at the terminals, is critical for various essential cell processes; for example, uridylation is implicated in tumorigenesis, proliferation, stem cell maintenance, and immune defense against viruses and retrotransposons. Ribosomal RNAs can be regulated by antisense ribosomal siRNAs (risiRNAs), which downregulate pre-rRNAs through the nuclear RNAi pathway in *Caenorhabditis elegans.* However, the biogenesis and regulation of risiRNAs remain obscure. Previously, we showed that 26S rRNAs are uridylated at the 3′-ends by an unknown terminal polyuridylation polymerase before the rRNAs are degraded by a 3′ to 5′ exoribonuclease SUSI-1(ceDIS3L2).

**Results:**

Here, we found that CDE-1, one of the three *C.elegans* polyuridylation polymerases (PUPs), is specifically involved in suppressing risiRNA production. CDE-1 localizes to perinuclear granules in the germline and uridylates Argonaute-associated 22G-RNAs, 26S, and 5.8S rRNAs at the 3′-ends. Immunoprecipitation followed by mass spectrometry (IP-MS) revealed that CDE-1 interacts with SUSI-1(ceDIS3L2). Consistent with these results, both CDE-1 and SUSI-1(ceDIS3L2) are required for the inheritance of RNAi.

**Conclusions:**

This work identified a rRNA surveillance machinery of rRNAs that couples terminal polyuridylation and degradation.

## Background

RNAs are extensively modified: 5′ termini are often capped, internal positions are altered on both ribose rings and bases, and 3′ termini receive untemplated nucleotides, which are referred to as tails. In eukaryotes, tails occur on most classes of RNAs, and they control RNA processing, stability, transport, and function. Terminal modification is critical in biology. For example, uridylation is implicated in tumorigenesis, proliferation, stem cell maintenance, and immune defense against viruses and retrotransposons [1–6]. The *C. elegans* genome encodes three polyuridylation polymerases (PUPs): *cde-1*/*pup-1*/*cid-1*, *pup-2*, and *pup-3* [7]. These PUPs may have distinct roles in different cellular contexts. *cde-1* is involved in the inheritance of RNAi, chromosome segregation, and antiviral defense [4, 8, 9]. CDE-1 functions with the RNA-dependent RNA polymerase (RdRP) EGO-1 and the Argonaute CSR-1 in the germline to affect chromosome segregation [10]. PUP-2/3 are the homologs of TUT4/7 (terminal uridylyl transferases 4/7) in mammals. PUP-2 targets the microRNA *let-7* and regulates the stability of LIN-28 [11]. The balance of CDE-1, PUP-2, and PUP-3 activities appears to ensure proper germline development in *C. elegans* [12].

Ribosome biogenesis is a very sophisticated multistep process, in which mistakes can occur at any step. Cells must carefully surveil the steps of the pre-rRNA processing and the assembly of ribosomal subunits. Misprocessed rRNAs are usually surveyed and degraded by multiple supervision machineries, including the exosome complex and the Trf4/Air2/Mtr4p polyadenylation (TRAMP) complex [13–15]. Aberrant RNAs are degraded by exosomes in a 3′-5′ exonucleolytic decay manner [16–18]. The exosome-independent exoribonuclease DIS3L2 plays a pivotal role in the 3′-5′ degradation of oligouridylated RNA fragments [19–23].

In addition to guiding erroneous rRNA degradation, antisense ribosomal siRNAs (risiRNAs) silence pre-rRNAs through the nuclear RNAi pathway to suppress the accumulation of erroneous rRNAs in *C. elegans* [20, 24–26]. Erroneous rRNAs are usually oligouridylated at the 3′-ends and then degraded by the exoribonuclease SUSI-1(ceDis3L2). However, it is unclear which terminal uridyltransferase performs the untemplated addition of the 3′-end uracil. Identifying which PUP is involved in the 3′-uridylation of erroneous rRNAs and how it is involved will further our understanding of the quality control mechanism of cellular nucleic acids.

Here, we found that CDE-1 is specifically involved in suppressing risiRNA production. CDE-1 localizes to perinuclear granules in the germline and uridylates both Argonaute-associated 22G-RNAs, 26S, and 5.8S rRNAs at the 3′-ends. Interestingly, we found that CDE-1 interacts with SUSI-1(ceDIS3L2). Both CDE-1 and SUSI-1(ceDIS3L2) are required for the inheritance of RNAi. Therefore, our findings are consistent with the model that CDE-1 suppresses generation of risiRNAs by uridylating rRNA and recruiting SUSI-1(ceDIS3L2) to the rRNA.

## Results

### Depletion of CDE-1 promotes risiRNA production

**Fig. 1:**
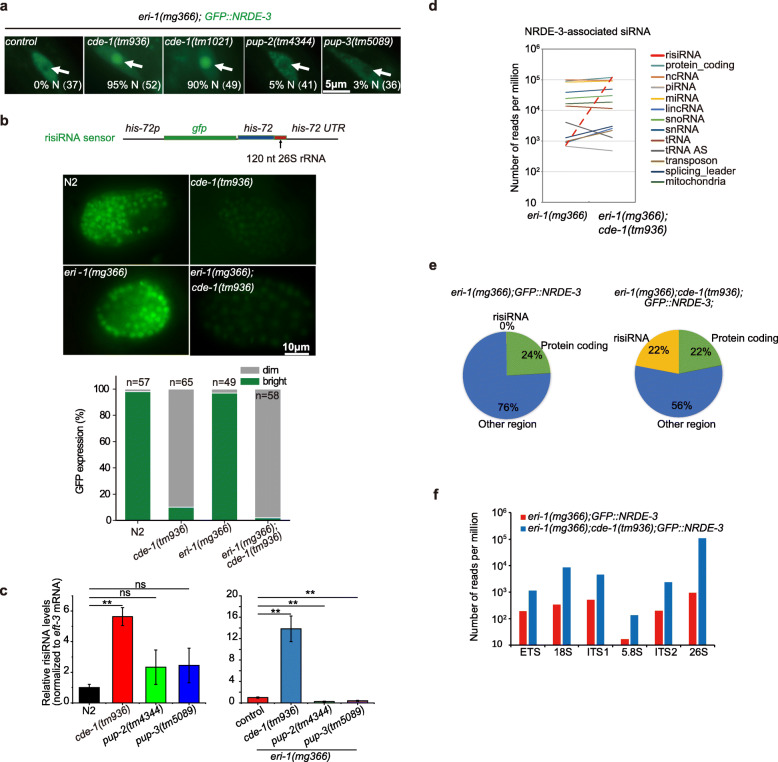
Antisense ribosomal siRNA (risiRNA) accumulats in *cde-1* mutant. **a** NRDE-3 localized to the nucleus in *eri-1(mg366);cde-1(tm936);GFP::NRDE-3* animals. Images show representative seam cells of the indicated animals. The percentage of animals with nuclear-enriched NRDE-3 in seam cells is indicated (% N). The number of scored animals is indicated in parentheses. White arrows, nucleus. **b** The risiRNA sensor was silenced in the *cde-1* mutant. (Top) Schematic representation of the risiRNA sensor. (Middle) Images of *C. elegans* embryos with the indicated genotypes expressing the risiRNA sensor at the ~ 300-cell stage. The levels of GFP expression were scored for each genotype in the bottom panel. **c** qRT-PCR analysis of risiRNA levels in indicated animals at the L3 stage. Data are presented as the mean ± SD (*n* = 3, biological replicates). ns, not significant. **P < 0.01 (two-tailed Student’s *t* test). **d**, **e** Deep sequencing of NRDE-3-associated siRNAs in indicated animals. The red dashed line indicates risiRNAs. **f** The number of risiRNAs targeting the each region of pre-rRNA transcription unit were analyzed

There are three RNA terminal uridylyltransferase genes, *cde-1*, *pup-2*, and *pup-3*, which are involved in RNA 3′-end uridylation in *C. elegans.* We previously showed that risiRNA was enriched in WAGO-4-bound siRNAs in *cde-1* mutants [8]. To further study the specificity and function of *cde-1* in risiRNA production, we used the *GFP::NRDE-3* transgene as a reporter. NRDE-3 is an Argonaute protein that transports siRNAs from the cytoplasm to the nucleus [27]. NRDE-3 localizes to the nucleus when it binds to siRNAs, but it accumulates in the cytoplasm when not bound to siRNA ligands. Disruption of the generation of endogenous siRNAs, for example, loss of function of *eri-1* mutant results in relocalization of NRDE-3 from the nucleus to the cytoplasm. We crossed *eri-1(mg366);GFP::NRDE-3* onto the *pup* mutant lines and found that the depletion of *cde-1*, but not *pup-2* or *pup-3*, was able to redistribute NRDE-3 from the cytoplasm to the nucleus (Fig. 1a). We generated a single copy transgene *CDE-1::mCherry* by Mos1-mediated single-copy insertion (MosSCI) technology. This transgene was able to rescue the *cde-1(tm936)* defects and redistribute NRDE-3 from the nucleus to the cytoplasm (Additional file 1: Fig. S1 A). To exclude the possibility that PUP-2 and PUP-3 act redundantly to suppress siRNA generation, we generated *pup-2;pup-3* double mutants. In the double mutants, NRDE-3 still accumulated in the cytoplasm (Additional file 1: Fig. S1 B). These data suggest that NRDE-3 was bound to newly generated siRNAs in *cde-1* mutants.

To test whether the NRDE-3-bound siRNAs in *cde-1* mutants contain risiRNA sequences, we used a risiRNA sensor expressing a *his-72p::gfp::his-72* reporter fused to the 26S rRNA sequence (Fig. 1b). The sensor was expressed in wild-type N2 and *eri-1(mg366)* animals but silenced in *cde-1(tm936)* mutants. Furthermore, we quantified the amount of risiRNA by quantitative real-time PCR analysis and found that risiRNAs were increased in *cde-1* mutants, but not in *pup-2* or *pup-3* mutants (Fig. 1c).

Last, we immunoprecipitated NRDE-3 and deep sequenced its associated small RNAs in *eri-1(mg366);GFP::NRDE-3* and *eri-1(mg366);cde-1(tm936);GFP::NRDE-3* animals in a 5′-phosphate-independent manner. Notably, the proportion of NRDE-3-bound risiRNAs increased approximately 164-fold in *eri-1(mg366);cde-1(tm936);GFP::NRDE-3* animals compared to the values observed in control animals (Fig. 1d, e). The abundance of risiRNAs targeting each rRNA region increased in *cde-1(tm936)* animals (Fig. 1f).

To search for the genetic requirements of risiRNA production in the *cde-1* mutants, we crossed *rrf-1*, *rrf-2*, and *rrf-3* lines onto the *eri-1(mg366);cde-1(tm936);GFP::NRDE-3* animals. RRF-1, RRF-2, and RRF-3 are RNA-dependent RNA polymerases that are important for the generation of 22G-RNAs in *C. elegans.* Consistent with previous results, the depletion of *rrf-1* and *rrf-2* together resulted in NRDE-3 being redistributed from the nucleus to the cytoplasm (Additional file 1: Fig. S1 C). In addition, the depletion of *rrf-1* and *rrf-2* together partially restored the fecundity of *eri-1;cde-1* animals (Additional file 1: Fig. S1 D).

Base on the above results, we conclude that *cde-1* likely acts as a suppressor of siRNA (*susi)* gene and suppresses the generation of risiRNAs.

### CDE-1 uridylates risiRNA

**Fig. 2:**
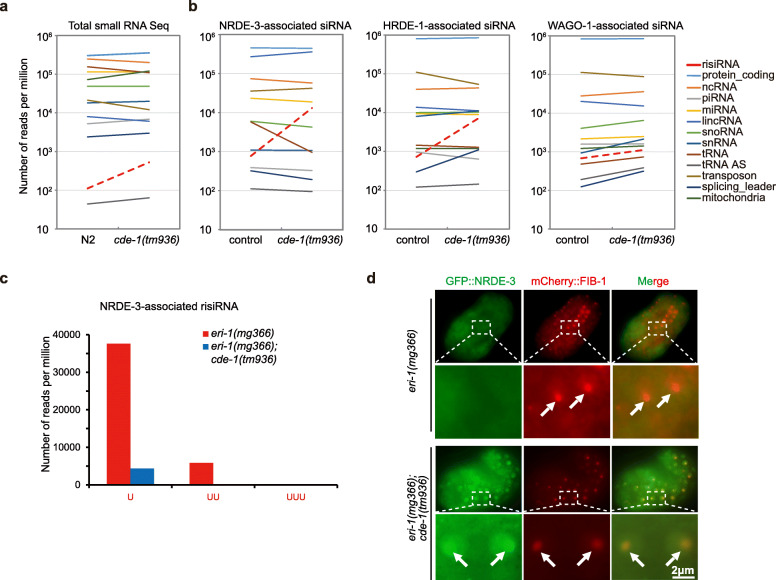
CDE-1 uridylates risiRNA. **a** Deep sequencing of total small RNAs of N2 and *cde-1* mutant. The red dashed line indicates risiRNAs. **b** Deep sequencing of Argonaute-associated siRNAs in the indicated animals. **c** Number of uridylated NRDE-3-associated risiRNAs in the indicated animals. **d** risiRNA elicited nucleolar accumulation of NRDE-3 in *cde-1* mutants. Images of *C. elegans* embryos of the indicated genotypes. White arrows, nucleolus

We first compared the small RNA expression profiles between wild-type and *cde-1* mutant animals. Small RNAs were isolated from young adult animals. Although the depletion of *cde-1* did not noticeably change the expression profile of different small RNA categories, risiRNAs were enriched 4.7 fold in *cde-1* mutant animals vs wild-type animals (Fig. 2a). We then immunoprecipitated GFP::NRDE-3 and deep sequenced the associated siRNAs. NRDE-3-bound risiRNAs were enriched 17 fold in *cde-1* mutant animals, compared to what was observed in control animals (Fig. 2b).

To test whether risiRNAs bind to Argonaute proteins in addition to NRDE-3, we analyzed HRDE-1 and WAGO-1-bound small RNAs in the young adult animals. HRDE-1 and WAGO-1 were immunoprecipitated from the control animals and the *cde-1(tm936)* mutant animals. Small RNAs were isolated and deep sequenced in the 5′-phosphate-independent method. In *cde-1* mutants, the amount of risiRNAs bound to HRDE-1 and WAGO-1 increased 9.9- and 1.6-fold, respectively, compared to those bound in wild-type animals (Fig. 2b). The NRDE-3-, HRDE-1-, and WAGO-1-bound small RNAs still exhibited the characteristics of 22G-RNAs, which is 22 nt in length and starts with 5′ guanidine in the mutants (Additional file 2: Fig. S2). A similar increase in risiRNA was observed in WAGO-4-bound risiRNAs in *cde-1* mutants [8]. CDE-1 adds untemplated uracil to the 3′-ends of CSR-1- and WAGO-4-bound siRNAs. We analyzed the NRDE-3-bound risiRNAs and found that there was a loss of the added untemplated uracil in *cde-1* mutants (Fig. 2c).

Small RNAs associate with NRDE-3 and guide NRDE-3 to the target nuclear nucleic acids. In the presence of risiRNA, NRDE-3 accumulated in the nucleoli of *cde-1* mutants (Fig. 2d). FIB-1 in *C. elegans* is encoded by an ortholog of the genes encoding human fibrillarin and *Saccharomyces cerevisiae* Nop1p [28, 29]. FIB-1 localizes to the nucleolus in embryos. This result suggested that NRDE-3 translocated to the nucleoli and risiRNAs may thereby silence rRNAs in the nucleoli due to double depletions of *cde-1* and *eri-1*.

### CDE-1 interacts with SUSI-1(ceDIS3L2) in the germline

**Fig. 3:**
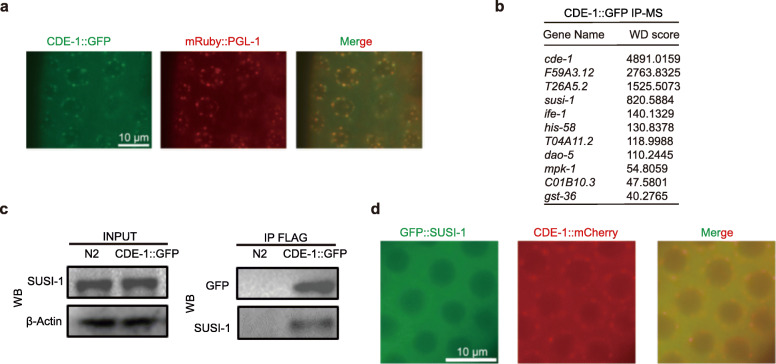
CDE-1 interacts with SUSI-1(ceDIS3L2) in the germline. **a** CDE-1 largely colocalized with the P-granule marker PGL-1. Images show the germline cells of young adult animals. **b** A summary of the top ten putative interacting proteins identified by CDE-1 immunoprecipitation followed by mass spectrometry. WD score: A scoring metrix for identifying high-confidence candidate interacting proteins (HCIPs) http://besra.hms.harvard.edu/ipmsmsdbs/cgi-bin/tutorial.cgi. **c** The protein-protein interaction of CDE-1 and SUSI-1(ceDIS3L2) was assayed by coimmunoprecipitation followed by western blotting with the indicated antibodies. **d** SUSI-1(ceDIS3L2) accumulated in the cytoplasm. Images of CDE-1 and SUSI-1 expression in the germline cells at young adult stage

To further understand the function of CDE-1, we constructed a GFP::3*×*FLAG tagged *cde-1p::CDE-1::GFP::3×FLAG* transgene (abbreviated as *CDE-1::GFP*) using the MosSCI technology. CDE-1 was expressed in the germline cells at all developmental stages (Additional file 3: Fig. S3 A). CDE-1 localized in the cytoplasm and accumulated in the perinuclear region exhibiting distinct foci in the germline of adult animals. We crossed the *CDE-1::GFP* strain with the P-granule marker strain *mRuby::PGL-1* and found that CDE-1 largely colocalized with the P-granule marker PGL-1 (Fig. 3a).

We further searched for proteins that interact with CDE-1. We used coimmunoprecipitation followed by mass spectrometry (IP-MS) to identify proteins that potentially interact with CDE-1. Strikingly, we identified SUSI-1(ceDis3L2) (Fig. 3B; Additional file 3: Fig. S3 B). SUSI-1(ceDIS3L2) is a 3′ to 5′ exoribonuclease that degrades oligouridylated RNAs. In *susi-1* mutants, both risiRNAs and oligouridylated rRNAs accumulated [20]. To confirm the protein-protein interaction between CDE-1 and SUSI-1(ceDIS3L2), we generated an antibody targeting SUSI-1(ceDIS3L2). CDE-1::GFP was immunoprecipitated by anti-FLAG antibody. Western blotting of the pelleted proteins with SUSI-1(ceDIS3L2) antiserum confirmed the protein-protein interaction between CDE-1 and SUSI-1(ceDIS3L2) in vivo (Fig. 3c). We then generated single-copy *3×FLAG::GFP::SUSI-1* and *CDE-1::mCherry* transgenes and found that SUSI-1(ceDIS3L2) accumulated in the cytoplasm of the germline cells (Fig. 3d).

Therefore, we conclude that CDE-1 and SUSI-1(ceDIS3L2) likely function as a protein complex to suppress risiRNAs production.

### CDE-1 is involved in uridylation of rRNA

**Fig. 4:**
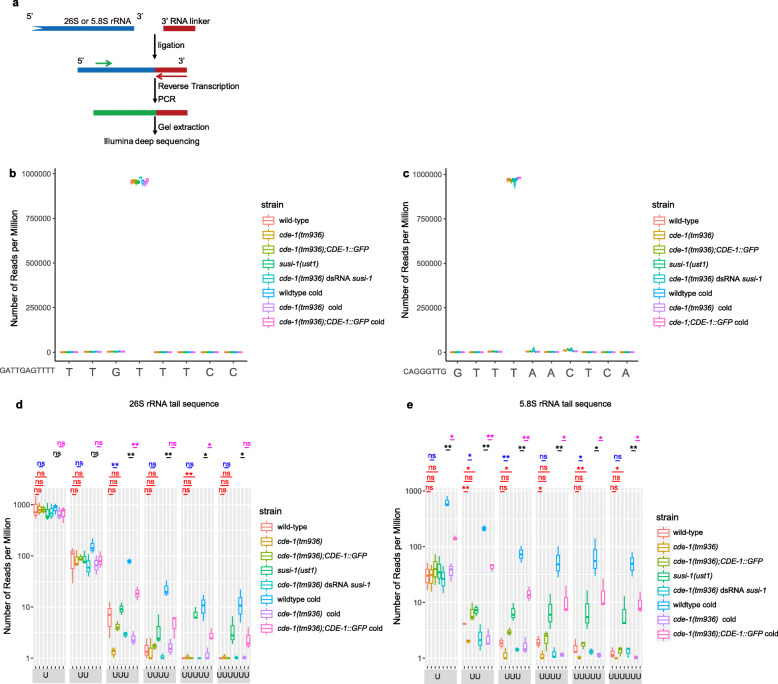
CDE-1 is involved in the 3′-end uridylation of 26S and 5.8S rRNAs. **a** A schematic of the rRNA tail-seq method. **b**, **d** Tail-seq data of 26S rRNAs from indicated animals at L3 stage. **c**, **e** Tail-seq data of 5.8S rRNAs from indicated animals at L3 stage. The numbers of reads with untemplated oligouridylation at the 3′-ends are indicated. Data are presented as the mean ± SD (*n* = 3, biological replicates). ns, not significant. *P < 0.05, **P < 0.01 (two-tailed Student’s *t* test)

Previously, we showed that SUSI-1(ceDIS3L2) degrades oligouridylated rRNAs and suppresses the production of risiRNA [20]. To examine whether CDE-1 uridylates rRNAs, a 3′ tail-seq method was used to detect the oligouridylation of rRNA at the 3′-tail in the indicated genotypes (Fig. 4a). Total RNA was isolated from L3-staged control and indicated animals, and then libraries were prepared by PCR amplification with a corresponding rRNA primer and a primer targeting the linker, which were followed by high-throughput sequencing (Fig. 4a).

The 3′-end of 26S rRNA was extensively modified by all four nucleotides compared to the annotated rRNA sequence [20]. We tried to detect the 3′-ends of 26S and 5.8S rRNA. We found that both 26S rRNA (Fig. 4b) and 5.8S rRNA (Fig. 4c) are well processed at the 3′-tails as the 3′-end nucleic acid of most RNAs is the same. Although we did not detect a dramatic change in the nontemplated addition of a single nucleotide, we observed a modest depletion of oligouridylation at the 3′-tail of 26S rRNA, comparing *cde-1(tm936)* to wild-type N2 animals at 20 °C. The depletion of oligouridylation of 26 rRNA can be rescued by a introduction of the *CDE-1::GFP* transgene (Fig. 4d). Previously, we have noticed that risiRNA was enriched in cold-shocked worms [20]. Here, we discovered a significant increase of oligouridylation at the 3′-tail of 26S rRNA, comparing wild-type N2 animals at 20 °C to cold-shocked N2 worms at 4 °C for 12 h. Meanwhile, *cde-1* mutation blocked the increase triggered by the cold shock treatment (Fig. 4d). We also noticed a significant increase of oligouridylation at the 3′-tail of 26S rRNA, comparing *susi-1(ust1)* to wild-type N2 animals at 20 °C (Fig. 4d). Furthermore, to uncover how CDE-1 and SUSI-1(ceDIS3L2) affect oligouridylation at the 3′-end of rRNA simultaneously, we knocked down *susi-1* in *cde-1(tm936)* mutants, as these two genes are too close on the genome to be genetically manipulated. We detected a significant decrease of *susi-1* mRNA level (Additional file 4: Fig. S4). However, there is no change of the oligouridylation at the 3′-tail of 26S rRNA, comparing to the untreated *cde-1(tm936)* worms.

As for 5.8S rRNA, we detected a dramatic change in the nontemplated addition of a single U nucleotide, which was accompanied by a significant increase of oligouridylation at the 3′-tail, comparing wild-type N2 animals at 20 °C to cold-shocked N2 worms at 4 °C. In the same way, depletion of *cde-1* blocked the increase of mono- or oligo-uridylation triggered by cold shock. Similarly, the change tendency of uridylation at the 3′-tail of 5.8S rRNA in the indicated genotype or under cold shock treatment is consistent with that of 26S rRNA (Fig. 4e).

Notably, it is obvious that CDE-1 prefers to add 3–6 Us to 3′-end of 26S rRNA and add 1–6 U(s) to 3′-tail of 5.8S rRNA, which may lead to degradation by SUSI-1(ceDIS3L2). This result indicates that SUSI-1(ceDIS3L2) may recognize, bind and process shorter oligoU uridylated rRNA, which is not consistent with the substrate preference (10–14 uridines or greater) of mouse Dis3l2 in the Lin28-let-7 pathway [22, 30].

Taken together, these data suggest that cold shock leads to uridylation at the 3′-tail of rRNA, and CDE-1 is involved in uridylating 26S and 5.8S rRNAs.

### SUSI-1(ceDIS3L2) is required for the inheritance of RNAi

**Fig. 5:**
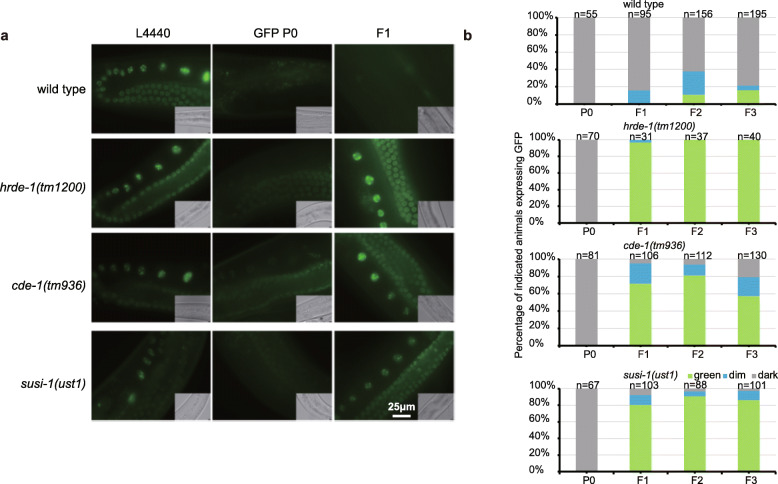
Both CDE-1 and SUSI-1(ceDIS3L2) are required for the inheritance of RNAi. **a** *mex-5p::GFP::H2B* transgenic animals were exposed to bacteria expressing *gfp* dsRNA. F1 embryos were isolated and grown on control bacteria in the absence of further *gfp* dsRNA treatment. GFP expression in the indicated animals was imaged in the germline and oocytes. **b** The percentage of P0 to F3 animals expressing GFP was counted

It was previously shown that CDE-1 is required for the inheritance of RNAi by uridylating WAGO-4-associated siRNAs [8, 31]. Since CDE-1 interacts with SUSI-1(ceDIS3L2), we then asked whether *susi-1* was also required for the inheritance of RNAi. We used a germline-expressed *mex-5p::GFP::H2B* (abbreviated as *GFP::H2B*) transgene as a reporter, which can inherit RNAi-induced gene silencing for multiple generations. Both *hrde-1* and *cde-1* were not required for exogenous *gfp* dsRNA to silence the *GFP::H2B* transgene in the parental generation, but they were essential for silencing in the F1 to F3 generations (Fig. 5). Similarly, *susi-1(ceDis3L2)* was not required for exogenous *gfp* dsRNA to silence the *GFP::H2B* transgene in the P0 generation but was necessary for silencing in F1 to F3 progeny. We conclude that *susi-1(ceDis3L2)* is required for the inheritance of RNAi.

## Discussion

Misprocessed rRNAs are usually detected and degraded by surveillance machinery during ribosome biogenesis [13, 14, 23, 32]. Previously, our lab identified a class of risiRNAs that downregulate pre-rRNAs through the nuclear RNAi pathway to suppress the accumulation of erroneous rRNAs. We identified a number of broadly conserved genes that are involved in rRNA processing and maturation. The depletion of these genes leads to an increase in risiRNAs. Thereafter, these genes are named suppressor of siRNA (*susi*) [20, 24]. Among them, SUSI-1(ceDIS3L2) plays a vital role in the 3′-5′ degradation of oligouridylated rRNA fragments [20]. In this work, we further found that CDE-1 uridylates the 3′-end of 26S and 5.8S rRNAs and may recruit SUSI-1(ceDIS3L2) through protein-protein interactions. Therefore, we conclude that *cde-1* is a new *susi* gene and suppresses the generation of risiRNAs.

**Fig. 6:**
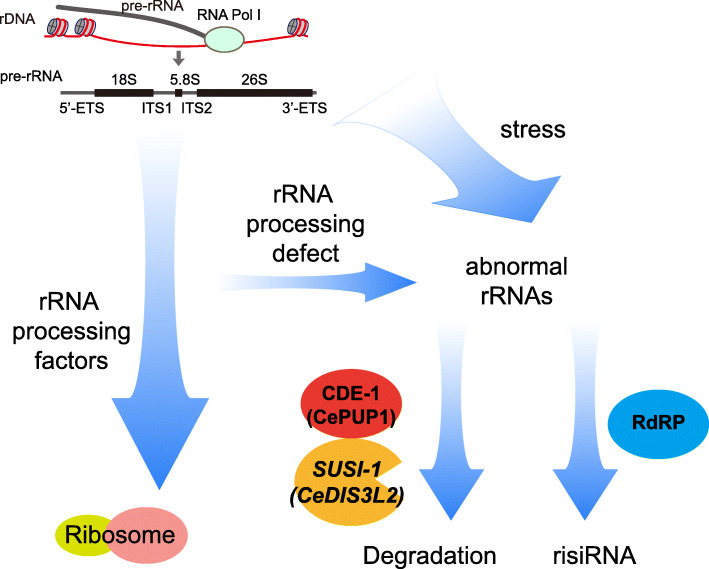
A working model of risiRNA biogenesis in *C. elegans*. The erroneous cellular rRNAs are scrutinized and suppressed through a number of mechanisms. Erroneous rRNAs are uridylated by CDE-1 and degraded by exoribonucleases such as SUSI-1(ceDis3L2). The disruption of CDE-1 or SUSI-1(ceDis3L2) results in the accumulation of erroneous rRNAs that thereafter recruit RdRPs to synthesize risiRNA. A risiRNA-mediated feedback loop silences rRNA expression through RNAi machinery and compensates for the disruption of the degradation of erroneous rRNA transcripts

Uridylation of the 3′-end of RNAs plays important functions in determining the fate of RNA [33, 34]. For example, uridylation is an intrinsic step in the maturation of noncoding RNAs, including the U6 spliceosomal RNA or mitochondrial guide RNAs in trypanosomes [35]. Uridylation can also switch specific miRNA precursors from a degradative to a processing mode. This switch depends on the number of uracils added and is regulated by the cellular context [36, 37]. However, the typical consequence of uridylation is accelerating the RNA degradation [21, 38]. In this work, we showed that CDE-1 can uridylate 26S and 5.8S rRNAs and recruit the 3′-5′ exoribonuclease SUSI-1(ceDIS3L2), which may further promote the degradation of oligouridylated rRNAs. In the absence of either CDE-1 or SUSI-1(ceDIS3L2), erroneous rRNAs will accumulate in cells, which thereafter recruit the RNA-dependent RNA polymerases, including RRF-1 and RRF-2, to initiate risiRNA production (Fig. 6). risiRNAs then bind to both nuclear and cytoplasmic Argonaute proteins and silence rRNAs through both nuclear and cytoplasmic RNAi machinery. Therefore, risiRNA and the RNAi machinery, together with exoribonucleases, act to avoid the accumulation of potentially harmful or unnecessary erroneous rRNA transcripts [14, 15, 17, 18, 32, 39, 40]. 3′-end modifications play important roles in regulating the stability of siRNAs via distinct mechanisms as well. For example, methylation of the 3′-end inhibits uridylation and correlates with increased steady state levels of small RNAs [41, 42]. In contrary, 3′ terminal uridylation may promote the degradation of siRNA [9, 43]. CDE-1 uridylates endogenous siRNAs and modulates their binding affinity to CSR-1 and WAGO-4 [8, 9]. PUP-2 has been reported to target *let-7* miRNA [11, 37]. Although PUP-3 has been validated as uridyl transferase, its targets are still unclear. Here, we found that CDE-1, but not PUP-2 or PUP-3, are engaged in suppressing risiRNA production. How these PUPs recognize their specific targets is still an enigma.

Additional questions remain as to how and why erroneous rRNAs could be recognized by CDE-1. Our previous work found that either the modification errors or processing errors of rRNAs trigger the generation of risiRNAs [24]. How these different kinds of errors are sensed and scrutinized is still unknown. Deciphering the intricate interaction network of CDE-1 or other TUTases is key to fully understanding the effect of RNA uridylation. In addition, CDE-1 was previously reported required for RNAi inheritance [8, 31]. The underlying mechanism remains unclear. Our previous data suggested that CDE-1 may mediate RNAi inheritance by regulating the binding of 22GRNAs to WAGO-4 [8]. Here, we found that CDE-1 interacted with SUSI-1(ceDIS3L2), another protein required for the inheritance of RNAi. Further elucidating the function of SUSI-1(ceDIS3L2) and CDE-1 will shed light on the mechanism of RNAi inheritance.

## Conclusions

RNA uridylation is a potent and widespread posttranscriptional regulator of gene expression. The untemplated terminal polyuridylation has a decisive impact on RNA’s fate. In this article, we further decipher the molecular mechanism of risiRNA generation in *C. elegans*. We provide genetic, cell biological, and biochemical evidence documenting the function of CDE-1, which uridylates rRNAs at 3′-ends. By using IP-MS technology, we found that CDE-1 interacts with SUSI-1(ceDIS3L2), a 3′-5′ exoribonuclease that we previously found to suppress risiRNA production. CDE-1 also uridylates Argonaute-associated risiRNAs. Interestingly, both CDE-1 and SUSI-1(ceDIS3L2) promote RNAi inheritance. In addition, this study sheds new light on a complicated surveillance network by combining uridylation, degradation, and RNAi-mediated gene silencing to maintain rRNA homeostasis.

## Methods

### Strains

Bistol strain N2 was used as the standard wild-type strain. All strains were grown at 20 °C unless otherwise specified. The strains used in this study were listed in Additional file 5: Table S1.

### Quantification of the subcellular location of NRDE-3

The subcellular localization of NRDE-3 was quantified as described previously [20]. Images were collected on a Leica DM4B microscope.

### Quantitative RT-PCR

RNA was isolated from the indicated animals and subjected to DNase I digestion (Thermo Fisher). cDNA was generated from the isolated RNA using a *GoScript*™ *Reverse Transcription System* (Promega) according to the vendor’s protocol. qPCR was performed using a MyIQ2 real-time PCR system (Bio-Rad) with an AceQ SYBR Green Master mix (Vazyme). The primers used in RT-qPCR were listed in Additional file 6: Table S2. *eft-*3 mRNA was used as an internal control for sample normalization. Data analysis was performed using a comparative threshold cycle (ΔΔCT) approach.

### Brood size

Synchronized L3 worms were individually placed onto fresh NGM plates, and the progeny numbers were scored.

### Construction of plasmids and transgenic strains

For *CDE-1::GFP*, a *cde-1* promoter and CDS region were PCR-amplified with the primers 5′-TACGACTCACTAGTGGGCAGgacgtgggacataaacgaagaaag-3′ and 5′-ATAGCTCCACCTCCACCTCCTTTGTTGTACGAGCGATGATAG-3′ from N2 genomic DNA. A *GFP::3×FLAG* region was PCR-amplified with the primers 5′-GGAGGTGGAGGTGGAGCTATGAGTAAAGGAGAAGAAC-3′ and 5′-TCACTTGTCATCGTCATCCT-3′ from plasmid pSG085. The CDE-1 3′ UTR (untranslated region) was PCR-amplified with the primers 5′- ACAAGGATGACGATGACAAGTAAattctctccacccattcac-3′ and 5′- CTACGTAATACGACTCACTTaactgatcggttgcttctctcac-3′ from N2 genomic DNA. A ClonExpress MultiS One-step Cloning Kit (Vazyme, C113–02) was used to insert the *CDE-1::GFP::3×FLAG* fusion gene into the pCFJ151 vector. The transgene was integrated into *C. elegans* chromosome II by the MosSCI method [44]. Using the same method, the *CDE-1::mCherry* fusion gene was integrated into *C. elegans* chromosome V.

The primers used for dual-sgRNA-directed CRISPR/Cas9-mediated *cde-1* gene deletion were 5′-TCCGGATAGTGATTACAATG-3′ and 5′-GGTATTATGTTGAACGACAT-3′.

For *3×FLAG::GFP::SUSI-1*, the predicted *susi-1* promoter was PCR-amplified with the primers 5′-TACGACTCACTAGTGGGCAGtatcagggagattctgctgtg-3′ and 5′- tcatggtctttgtagtccatACTTTCAACTGCTGACATctag-3′ from N2 genomic DNA. The *3×FLAG::GFP* coding region was PCR amplified from plasmid pSG085 with the primers 5′-AGCTCTTCCTATGGACTACAAAGACCATGAC-3′ and 5′- ATAGCTCCACCTCCACCTCCTTTGTATAGTTCATCCATGCC-3′. The *susi-1* coding region and the predicted 3′ UTR were then amplified by PCR from N2 genomic DNA with primers 5′-AAGGAGGTGGAGGTGGAGCTATGTCAGCAGTTGAAAGTCCCG-3′ and 5′-CTACGTAATACGACTCACTTGTGTGGATTAACACAGCCAATTG-3′ from N2 genomic DNA. The ClonExpress MultiS One-step Cloning Kit (Vazyme, C113-02) was used to insert the *3×FLAG::GFP::SUSI-1* fusion gene into the pCFJ151 vector. The transgene was integrated into *C. elegans* chromosome II by the MosSCI system.

### RNA immunoprecipitation (RIP)

RIP experiments were performed as previously described with hypochlorite-isolated embryos of indicated animals [20]. The embryos were sonicated in lysis buffer (20 mM Tris-HCl (pH 7.5), 200 mM NaCl, 2.5 mM MgCl_2_, and 0.5% NP-40), precleared with protein G-agarose beads (Roche), and incubated with anti-FLAG M2 agarose beads (Sigma #A2220). The beads were washed extensively, and 3*×*FLAG::GFP-tagged protein and associated RNAs were eluted with 100 μg/mL 3*×*FLAG peptide (Sigma). The eluates were incubated with TRIzol reagent (Invitrogen), which was followed by isopropanol precipitation. Then, small RNAs were quantified by deep sequencing.

### Deep sequencing of small RNAs and bioinformatic analysis

Total RNAs and the Argonaute-associated RNAs were isolated from the indicated animals and subjected to small RNA deep sequencing using an Illumina platform (Novogene Bioinformatics Technology Co., Ltd.), as previously described [20].

For Argonaute-associated RNAs, synchronized worms were sonicated in sonication buffer (20 mM Tris-HCl,pH 7.5, 200 mM NaCl, 2.5 mM MgCl_2_, and 0.5% NP40). The eluates were incubated with TRIzol reagent and then precipitated with isopropanol. The precipitated RNA was treated with FastAP Thermosensitive Alkaline Phosphatase (Thermo Scientific), re-extracted with TRIzol, and treated with T4 Polynucleotide Kinase (T4 PNK, Thermo Scientific) in the presence of 1 mM ATP before library construction.

Small RNAs were subjected to deep sequencing using an Illumina platform (Novogene Bioinformatics Technology Co., Ltd.). Briefly, small RNAs ranging from 18 to 30 nt were gel-purified and ligated to a 3′ adaptor (5′-pUCGUAUGCCGUCUUCUGCUUGidT-3′; p, phosphate; idT, inverted deoxythymidine); and a 5′ adaptor (5′-GUUCAGAGUUCUACAGUCCGACGAUC-3′), respectively. The ligation products were gel-purified, reverse transcribed, and amplified using an Illumina sRNA primer set (5′-CAAGCAGAAGACGGCATACGA-3′; 5′-AATGATACGGCGACCACCGA-3′). The samples were then sequenced using an Illumina HiSeq platform.

The Illumina-generated raw reads were first filtered to remove adaptors, low-quality tags, and contaminants to obtain clean reads by Novogene. Clean reads ranging from 18 to 30 nt were mapped to the transcriptome assembly WS243 using Bowtie2 with default parameters. The number of reads targeting each transcript were counted by custom Perl scripts. The number of total reads mapped to the genome minus the number of total reads corresponded to sense rRNA transcripts (5S, 5.8S, 18S, and 26S), which was used as the normalization number, to exclude the possible degradation fragments of sense rRNAs.

### Proteomic analysis

Proteomic analysis was conducted as previously described [45]. Briefly, mixed-stage transgenic worms expressing CDE-1::GFP were resuspended in equal volumes of 2*×* lysis buffer (50 mM Tris-HCl pH 8.0, 300 mM NaCl, 10% glycerol, 1% Triton X-100, Roche®cOmplete EDTA-free Protease Inhibitor Cocktail, 10 mM NaF, and 2 mM Na_3_VO_4_), and lysed in a FastPrep-24 5G homogenizer. The lysate supernatant was incubated with anti-GFP antibody, which was linked to beads, for 1 h at 4 °C. The beads were then washed three times with cold lysis buffer. The GFP immunoprecipitates were eluted with chilled elution buffer (100 mM glycine-HCl, pH 2.5). Approximately 1/8 of the eluates were subjected to western blotting analysis. The rest of the eluates were precipitated with TCA or cold acetone and dissolved in 100 mM Tris (pH 8.5), with 8 M urea. The proteins were reduced with TCEP, alkylated with IAA, and finally digested with trypsin at 37 °C overnight. LC-MS/MS analysis of the resulting peptides and MS data processing approaches were conducted as previously described [46]. A WD scoring matrix was used to identify high-confidence candidate interacting proteins.

### Coimmunoprecipitation analysis

The lysates of transgenic worms were prepared using RIP lysis buffer (50 mM Tris (pH 7.4), 150 mM NaCl, 1% NP-40, 0.1% SDS, 1 mM EDTA, 0.5% sodium deoxycholate, and protease inhibitors (Thermo)). Immunoprecipitations with anti-FLAG® M2 affinity gel (a2220, Sigma) or agarose beads (ab193255, Abcam) with anti-GFP antibody (ab290, Abcam) and anti-SUSI-1 antibody (lot number 20121105, Abmart) were performed at 4 °C overnight. Protein complexes were eluted by boiling in 2× SDS loading buffer. Anti-GFP, anti-SUSI-1 and anti-Actin (Servicebio GB12001) antibodies that were used for western blots were diluted to 1:2000, 1:500, and 1:5000, respectively.

### rRNA 3′ tail-seq

rRNA tail-seq was conducted as described previously [20]. Briefly, total RNA were extracted from L3 larva, digested by DNase I, and then ligated to the following 3′ RNA linkers with T4 RNA ligase (Thermo #EL0021) (1 μg total RNA, 2 μL 3′ RNA linker (10 μM), 1 μL 10× T4 RNA ligation buffer, 2 μL T4 RNA ligase) by incubating at 37 °C for 30 min.

3′ RNA linker-1:

5′-pGATCCACACTCGGGCACCAAGGATTTAACCGCGAATTCCAGC-NH2–3′ (the underlined sequence served as a barcode for sample labeling).

3′ RNA linker-2:

5′-pCGACACACTCGGGCACCAAGGATTTAACCGCGAATTCCAGC-NH2–3′.

3′ RNA linker-3:

5′-pGTACCACACTCGGGCACCAAGGATTTAACCGCGAATTCCAGC-NH2–3′.

The RNAs were reverse transcribed with the following primer:

Universal 3′ linker RT:

5′-GCTGGAATTCGCGGTTAAATCCTTGGTGCCCGAGTGT-3′. The cDNAs were PCR amplified (16 cycles) with the primers 26S rRNA-F: 5′-CAGATCACTCTGGTTCAATGTC-3′ and universal 3′ linker RT primer, or with the primers 5.8S rRNA-F: 5′-GGTTGCATCGAGTATCGATGAA-3′ and universal 3′ linker RT primer, gel purified and then deep sequenced using an Illumina platform, according to the manufacturer′s instructions, by Novogene (Beijing, China). The number of reads with distinct 3′-end modifications was counted by custom Perl scripts. Three biological replicates were conducted in each genotype or treatment.

### RNAi inheritance assay

Synchronized L1 animals of the indicated genotypes were exposed to bacteria expressing *gfp* dsRNA. F1 embryos were collected by hypochlorite/NaOH treatment and grown on OP50 bacteria. The GFP expression levels in both the parental generation (P0) and the progeny (F1–F3) were visualized and scored. Images were collected with a Leica DM4B microscope system.

### Statistics

Bar graphs with error bars represent the mean and SD. All of the experiments were conducted with independent *C. elegans* animals for the indicated N replicates. Statistical analysis was performed with two-tailed Student’s *t* tests or unpaired Wilcoxon tests. The threshold for Student’s *t* test *P* values was set to 0.05 or 0.01.

## Supplementary information

**Additional file 1: Figure S1.** risiRNA accumulats in *cde-1* mutant and requires *rrf-1* and *rrf-2*. (A) → *CDE-1::mCherry* was able to redistribute NRDE-3 from the nucleus to the cytoplasm in *cde-1(tm936)* mutants. The germline and the seam cells of indicated animals at L3 stage were shown. White dashed line shows the border of germline. White arrows, nucleus. (B) → The depletion of *pup-2* and *pup-3* together was not able to redistribute NRDE-3 from the cytoplasm to the nucleus. The seam cells of indicated animals were shown. The numbers indicated the percentage of animals with nuclear enriched NRDE-3 in seam cells. The number of scored animals is indicated in the parentheses. White arrows, nucleus. (C) → *rrf-1* and *rrf-2* were required for NRDE-3 nuclear localization when *cde-1* is not functional. Images of the representative seam cells were shown. The numbers indicated the percentage of animals with nuclear enriched NRDE-3 in seam cells. The number of scored animals is indicated in the parentheses. White arrows, nucleus. (D) → The depletion of *rrf-1* and *rrf-2* partially restored the fecundity of *eri-1(mg366);cde-1(tm936)* animals. Data are presented as the mean ± SD (*n* = 3, biological replicates). **P < 0.01 (two-tailed Student’s t test).

**Additional file 2: Figure S2.** Size distribution and 5′ end nucleotide preference of siRNAs identified by deep sequencing. (A) NRDE-3-, (B) HRDE-1-, and (C) WAGO-1-bound small RNAs in indicated animals were deep sequenced. Size distribution and 5′ end nucleotide preference were analyzed.

**Additional file 3: Figure S3.** CDE-1 interacts with SUSI-1(ceDIS3L2) in the germline. (A) → CDE-1::GFP was visualized by fluorescent microscopy at indicated developmental stages. White dashed lines show the borders of embryos or germlines at indicated developmental stages. Green dashed lines indicate P cell lineage at embryo stage. (B) → Western blotting analysis of CDE-1::GFP was performed after GFP immunoprecipitation. (C) → SUSI-1(ceDIS3L2) accumulated in the cytoplasm. Images of CDE-1::mCherry (largely colocalized with the P-granule, the upper panel) and GFP::SUSI-1 (in cytoplasm, the upper and lower panels) expression in the germline cells at young adult stage.

**Additional file 4: Figure S4.** qRT-PCR analysis of *susi-1* mRNA levels in indicated animals at the L3 stage. Data are presented as the mean ± SD (n = 3, biological replicates). *P < 0.05 (two-tailed Student’s t test).

**Additional file 5: Table S1.** Strains used in this work.

**Additional file 6: Table S2.** Primers used for quantitative real-time PCR analysis.

## Data Availability

All raw and normalized sequencing data have been deposited to Gene Expression Omnibus under submission number GSE139530 [47] and GSE156000 [48].
