# Supplementary figures and images for: CDE-1 suppresses the production of risiRNA by coupling polyuridylation and degradation of rRNA
Source: BMC Biol. 2020 Sep 4;18:115. doi: 10.1186/s12915-020-00850-z (PMC7472701; doi:10.1186/s12915-020-00850-z)

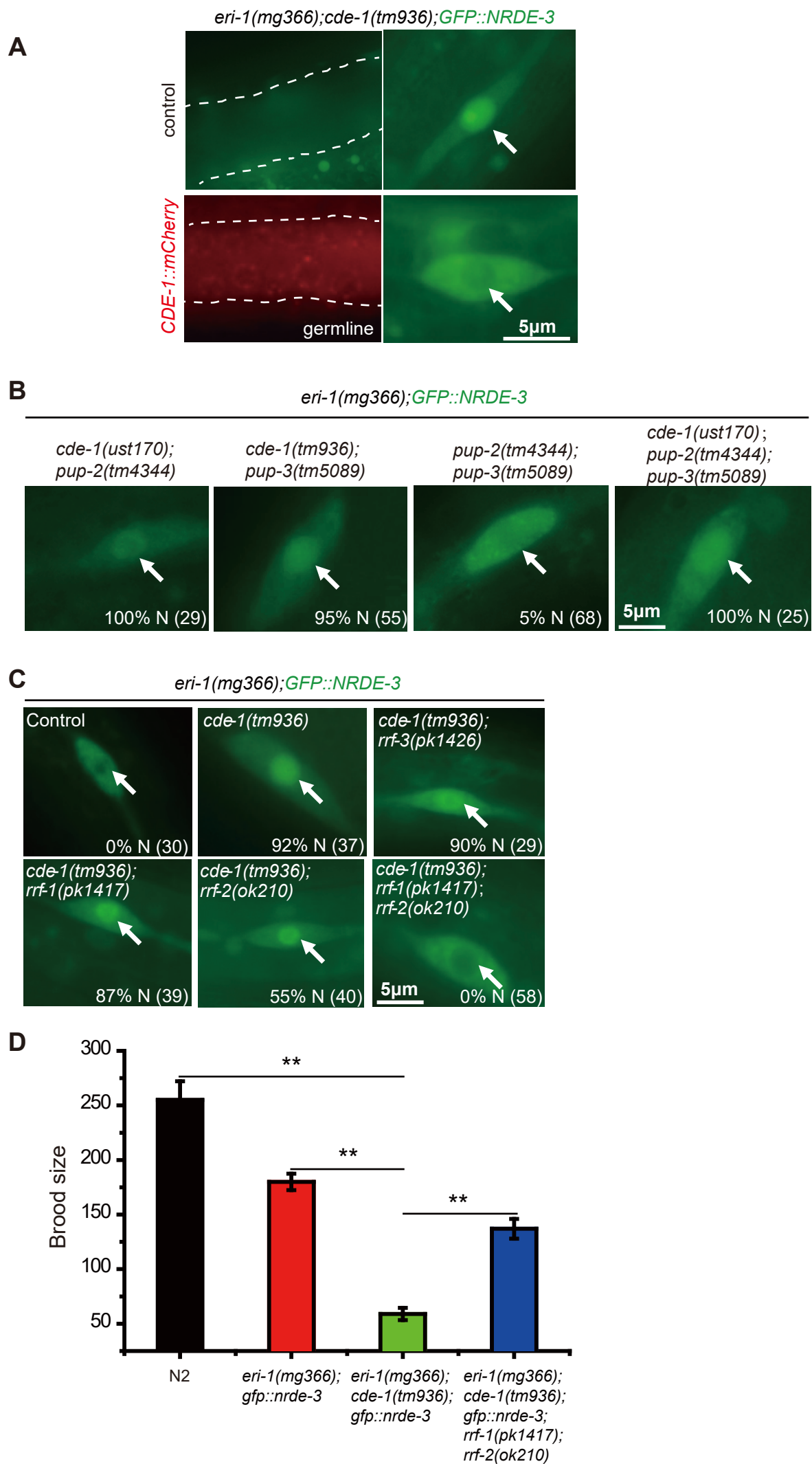

Supplement: Supplementary file 1 — Additional file 1: Figure S1. risiRNA accumulats in cde-1 mutant and requires rrf-1 and rrf-2. (A) → CDE-1::mCherry was able to redistribute NRDE-3 from the nucleus to the cytoplasm in cde-1(tm936) mutants. The germline and the seam cells of indicated animals at L3 stage were shown. White dashed line shows the border of germline. White arrows, nucleus. (B) → The depletion of pup-2 and pup-3 together was not able to redistribute NRDE-3 from the cytoplasm to the nucleus. The seam cells of indicated animals were shown. The numbers indicated the percentage of animals with nuclear enriched NRDE-3 in seam cells. The number of scored animals is indicated in the parentheses. White arrows, nucleus. (C) → rrf-1 and rrf-2 were required for NRDE-3 nuclear localization when cde-1 is not functional. Images of the representative seam cells were shown. The numbers indicated the percentage of animals with nuclear enriched NRDE-3 in seam cells. The number of scored animals is indicated in the parentheses. White arrows, nucleus. (D) → The depletion of rrf-1 and rrf-2 partially restored the fecundity of eri-1(mg366);cde-1(tm936) animals. Data are presented as the mean ± SD (n = 3, biological replicates). **P < 0.01 (two-tailed Student’s t test). [file 12915_2020_850_MOESM1_ESM.pdf]

**A**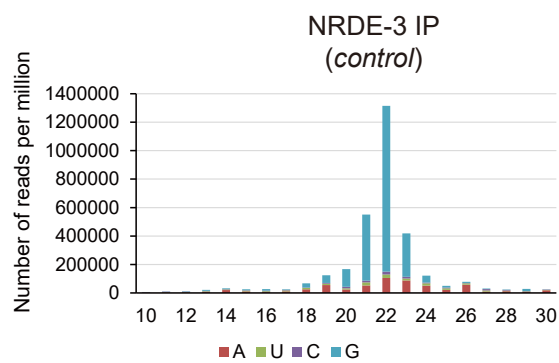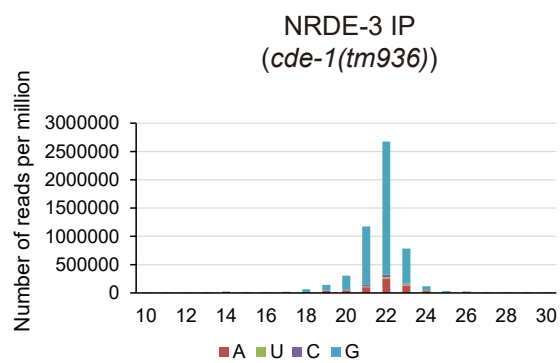**B**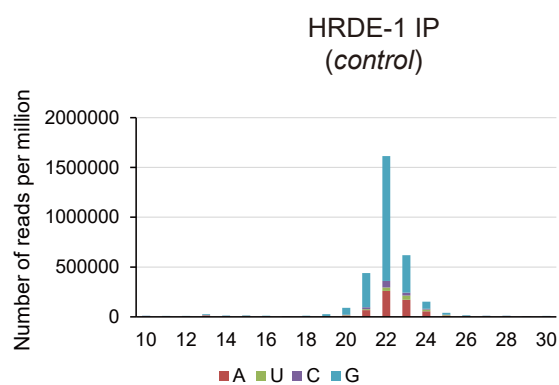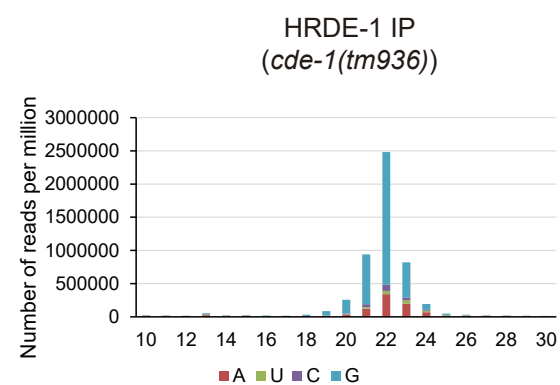**C**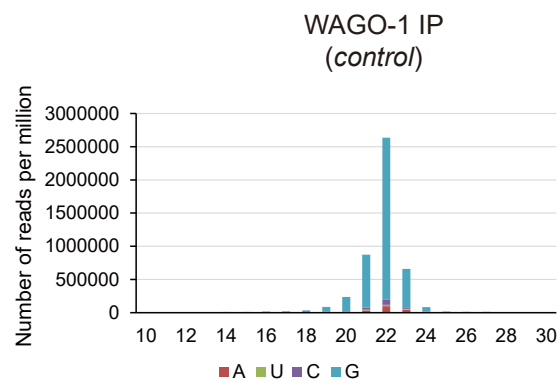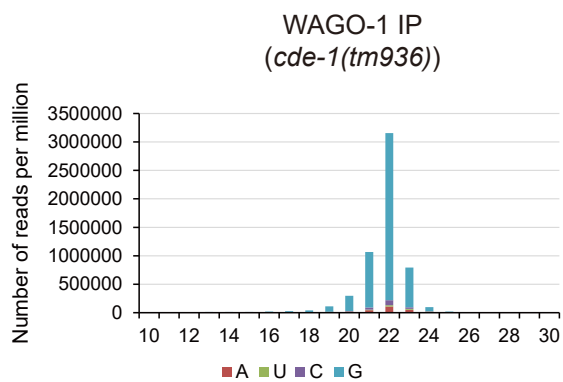**Figure S2**

Supplement: Supplementary file 2 — Additional file 2: Figure S2. Size distribution and 5′ end nucleotide preference of siRNAs identified by deep sequencing. (A) NRDE-3-, (B) HRDE-1-, and (C) WAGO-1-bound small RNAs in indicated animals were deep sequenced. Size distribution and 5′ end nucleotide preference were analyzed. [file 12915_2020_850_MOESM2_ESM.pdf]

**A**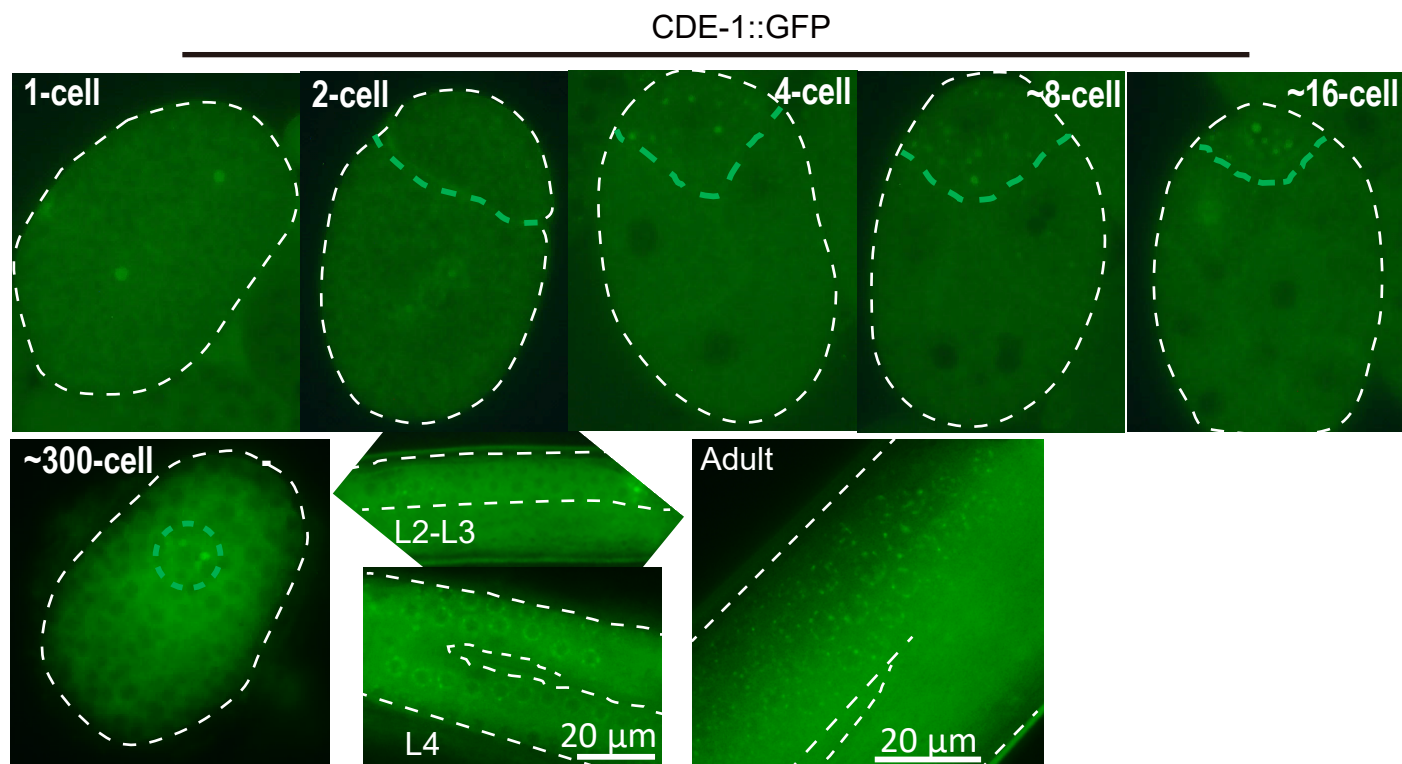**B**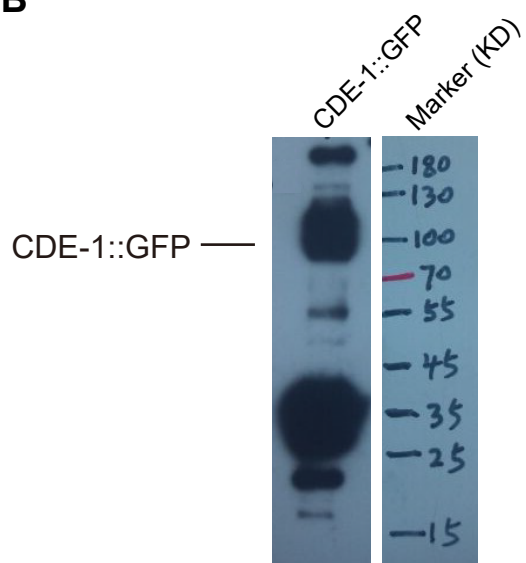**C**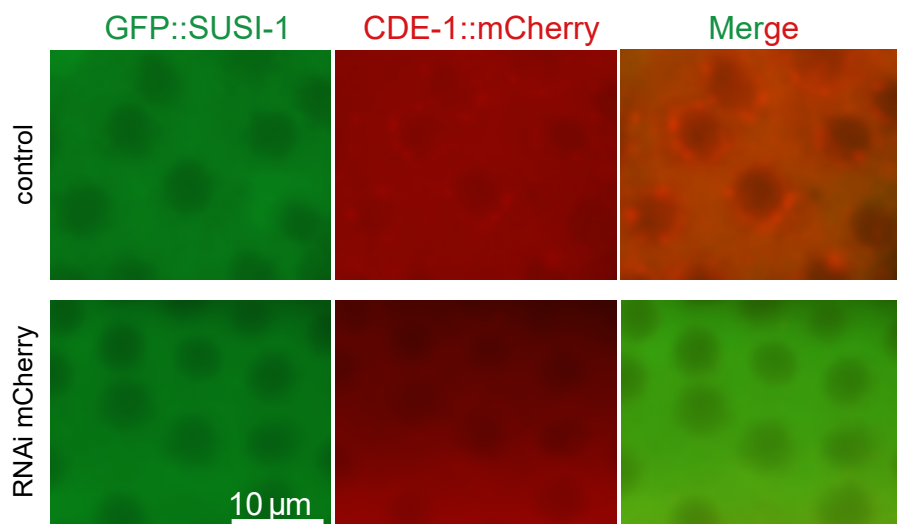**Figure S3**

Supplement: Supplementary file 3 — Additional file 3: Figure S3. CDE-1 interacts with SUSI-1(ceDIS3L2) in the germline. (A) → CDE-1::GFP was visualized by fluorescent microscopy at indicated developmental stages. White dashed lines show the borders of embryos or germlines at indicated developmental stages. Green dashed lines indicate P cell lineage at embryo stage. (B) → Western blotting analysis of CDE-1::GFP was performed after GFP immunoprecipitation. (C) → SUSI-1(ceDIS3L2) accumulated in the cytoplasm. Images of CDE-1::mCherry (largely colocalized with the P-granule, the upper panel) and GFP::SUSI-1 (in cytoplasm, the upper and lower panels) expression in the germline cells at young adult stage. [file 12915_2020_850_MOESM3_ESM.pdf]

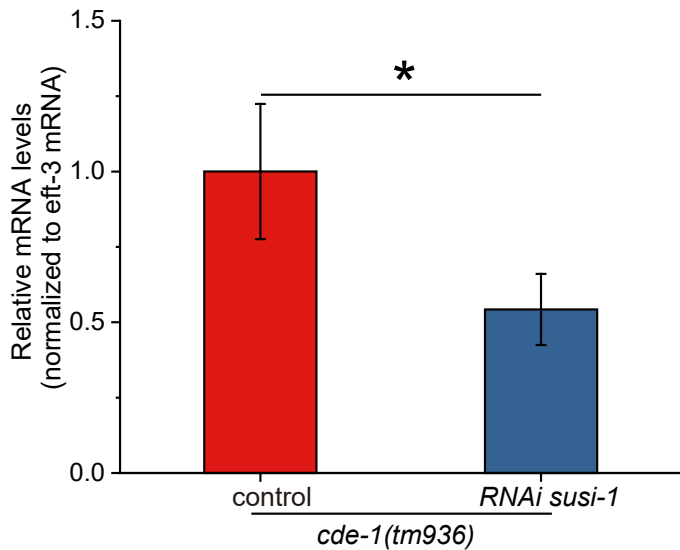

**Figure S4**

Supplement: Supplementary file 4 — Additional file 4: Figure S4. qRT-PCR analysis of susi-1 mRNA levels in indicated animals at the L3 stage. Data are presented as the mean ± SD (n = 3, biological replicates). *P < 0.05 (two-tailed Student’s t test). [file 12915_2020_850_MOESM4_ESM.pdf]
