# Supplementary material for: CDE-1 suppresses the production of risiRNA by coupling polyuridylation and degradation of rRNA
Source: BMC Biol. 2020 Sep 4;18:115. doi: 10.1186/s12915-020-00850-z (PMC7472701; doi:10.1186/s12915-020-00850-z)
Supplement: Supplementary file 5 — Additional file 5: Table S1. Strains used in this work. [file 12915_2020_850_MOESM5_ESM.docx]

Table S1: Strains used in this work.

| Genotype |
| --- |
| N2 |
| *eri-1(mg366)* |
| *eri-1(mg366);nrde-3p::GFP::NRDE-3(ggISI)* |
| *cde-1(tm936)* |
| *cde-1(tm1021)* |
| *eri-1(mg366);cde-1(tm936)* |
| *eri-1(mg366);cde-1(tm936); nrde-3p::GFP::NRDE-3(ggISI)* |
| *eri-1(mg366);cde-1(tm1021); nrde-3p::GFP::NRDE-3(ggISI)*  *cde-1p::CDE-1::mCherry(ustIS105)* |
| *eri-1(mg366);cde-1(tm936); nrde-3p::GFP::NRDE-3(ggISI); cde-1p::CDE-1::mCherry* |
| *pup-2(tm4344)* |
| *pup-3(tm5089)* |
| *eri-1(mg366);pup-2(tm4344); nrde-3p::GFP::NRDE-3(ggISI)* |
| *eri-1(mg366);pup-3(tm5089); nrde-3p::GFP::NRDE-3(ggISI)* |
| *eri-1(mg366);pup-2(tm4344);pup-3(tm5089); nrde-3p::GFP::NRDE-3(ggISI)* |
| *eri-1(mg366);cde-1(tm936);pup-3(tm5089); nrde-3p::GFP::NRDE-3(ggISI)*  *eri-1(mg366);cde-1(ust170); pup-2(tm4344); nrde-3p::GFP::NRDE-3(ggISI)*  *eri-1(mg366);cde-1(ust170); pup-2(tm4344); pup-3(tm5089); nrde-3p::GFP::NRDE-3(ggISI)* |
| *susi-1(ust1)* |
| *eri-1(mg366); susi-1(ust1); nrde-3p::GFP::NRDE-3(ggISI)* |
| *control_sensor(ustIS38)* |
| *risiRNA_sensor(ustIS37)* |
| *risiRNA_sensor(ustIS37);eri-1(mg366)* |
| *risiRNA_sensor(ustIS37);cde-1(tm936)* |
| *risiRNA_sensor(ustIS37);eri-1(mg366);cde-1(tm936)* |
| *mCherry::FIB-1(ustIS36)* |
| *eri-1(mg366); nrde-3p::GFP::NRDE-3(ggISI);mCherry::FIB-1(ustIS36)* |
| *eri-1(mg366); nrde-3p::GFP::NRDE-3(ggISI);mCherry::FIB-1(ustIS36);cde-1(tm936)* |
| *eri-1(mg366);cde-1(tm936); nrde-3p::GFP::NRDE-3(ggISI);rrf-1(pk1417)* |
| *eri-1(mg366);cde-1(tm936); nrde-3p::GFP::NRDE-3(ggISI);rrf-2(ok210)* |
| *eri-1(mg366);cde-1(tm936); nrde-3p::GFP::NRDE-3(ggISI);rrf-3(pk1426)* |
| *eri-1(mg366);cde-1(tm936); nrde-3p::GFP::NRDE-3(ggISI);rrf-1(pk1417);rrf-2(ok210)* |
| *hrde-1p::GFP::HRDE-1(ustIS68)* |
| *cde-1(tm936); hrde-1p::GFP::HRDE-1(ustIS68)* |
| *wago-1p::GFP::WAGO-1(ustIS106)* |
| *cde-1(tm936); wago-1p::GFP::WAGO-1(ustIS106)* |
| *cde-1p::CDE-1::GFP(ustIS107)* |
| *dpy-30p::mRuby::PGL-1(hjSi396)* |
| *cde-1p::CDE-1::GFP(ustIS107); dpy-30p::mRuby::PGL-1(hjSi396)* |
| *susi-1p::GFP::SUSI-1(ustIS108)* |
| *cde-1p::CDE-1::mCherry(ustIS105); susi-1p::GFP::SUSI-1(ustIS108)* |
| *mex-5p::GFP::H2B(ustIS45)* |
| *hrde-1(tm1200); mex-5p::GFP::H2B(ustIS45)* |
| *cde-1(tm936); mex-5p::GFP::H2B(ustIS45)* |
| *susi-1(ust1); mex-5p::GFP::H2B(ustIS45)* |
