# Supplementary material for: CDE-1 suppresses the production of risiRNA by coupling polyuridylation and degradation of rRNA
Source: BMC Biol. 2020 Sep 4;18:115. doi: 10.1186/s12915-020-00850-z (PMC7472701; doi:10.1186/s12915-020-00850-z)
Supplement: Supplementary file 6 — Additional file 6: Table S2. Primers used for quantitative real-time PCR analysis. [file 12915_2020_850_MOESM6_ESM.docx]

**Table S2 Primers used for quantitative real-time PCR analysis**

| *eft-3* RT F | ACTTGATCTACAAGTGCGGAGGA |
| --- | --- |
| *eft-3* RT R | AAAGATCCCTTACCCATCTCCTG |
| risiRNA RT | GTCGTATCCAGTGCGTGTCGTGGAGTCGGCAATTGCACTGGATACGATGTCGGG |
| risiRNA qRT F | GTGCGTGTCGTGGAGTCG |
| risiRNA qRT R  *susi-1* mRNA F  *susi-1* mRNA R | TGTCGGGAGGCATCTCTATCTC  CTGCCGCTACAGTCAAAACC  CCTTTGATATGTATTCGGTGAAGAT |
